# Meticillin-resistant *Staphylococcus aureus* with a novel *mecA* homologue in human and bovine populations in the UK and Denmark: a descriptive study

**DOI:** 10.1016/S1473-3099(11)70126-8

**Published:** 2011-08

**Authors:** Laura García-Álvarez, Matthew TG Holden, Heather Lindsay, Cerian R Webb, Derek FJ Brown, Martin D Curran, Enid Walpole, Karen Brooks, Derek J Pickard, Christopher Teale, Julian Parkhill, Stephen D Bentley, Giles F Edwards, E Kirsty Girvan, Angela M Kearns, Bruno Pichon, Robert LR Hill, Anders Rhod Larsen, Robert L Skov, Sharon J Peacock, Duncan J Maskell, Mark A Holmes

**Affiliations:** a Department of Veterinary Medicine, University of Cambridge, UK; b The Wellcome Trust Sanger Institute, Wellcome Trust Genome Campus, Cambridge, UK; c Health Protection Agency, Addenbrooke's Hospital, Cambridge, UK; d Veterinary Laboratories Agency, Shrewsbury, UK; e Scottish MRSA Reference Laboratory, NHS Greater Glasgow and Clyde, Stobhill Hospital, Glasgow, UK; f Microbiology Services Division, Health Protection Agency, London, UK; g Department of Antimicrobial Surveillance and Research, Statens Serum Institut, Copenhagen, Denmark; h Department of Medicine, University of Cambridge, Addenbrooke's Hospital, Cambridge, UK

## Abstract

**Background:**

Animals can act as a reservoir and source for the emergence of novel meticillin-resistant *Staphylococcus aureus* (MRSA) clones in human beings. Here, we report the discovery of a strain of *S aureus* (LGA251) isolated from bulk milk that was phenotypically resistant to meticillin but tested negative for the *mecA* gene and a preliminary investigation of the extent to which such strains are present in bovine and human populations.

**Methods:**

Isolates of bovine MRSA were obtained from the Veterinary Laboratories Agency in the UK, and isolates of human MRSA were obtained from diagnostic or reference laboratories (two in the UK and one in Denmark). From these collections, we searched for *mecA* PCR-negative bovine and human *S aureus* isolates showing phenotypic meticillin resistance. We used whole-genome sequencing to establish the genetic basis for the observed antibiotic resistance.

**Findings:**

A divergent *mecA* homologue (*mecA*_LGA251_) was discovered in the LGA251 genome located in a novel staphylococcal cassette chromosome *mec* element, designated type-XI SCC*mec*. The *mecA*_LGA251_ was 70% identical to *S aureus mecA* homologues and was initially detected in 15 *S aureus* isolates from dairy cattle in England. These isolates were from three different multilocus sequence type lineages (CC130, CC705, and ST425); *spa* type t843 (associated with CC130) was identified in 60% of bovine isolates. When human *mecA*-negative MRSA isolates were tested, the *mecA*_LGA251_ homologue was identified in 12 of 16 isolates from Scotland, 15 of 26 from England, and 24 of 32 from Denmark. As in cows, t843 was the most common *spa* type detected in human beings.

**Interpretation:**

Although routine culture and antimicrobial susceptibility testing will identify *S aureus* isolates with this novel *mecA* homologue as meticillin resistant, present confirmatory methods will not identify them as MRSA. New diagnostic guidelines for the detection of MRSA should consider the inclusion of tests for *mecA*_LGA251_.

**Funding:**

Department for Environment, Food and Rural Affairs, Higher Education Funding Council for England, Isaac Newton Trust (University of Cambridge), and the Wellcome Trust.

## Introduction

*Staphylococcus aureus* causes a wide range of diseases in human beings, from minor skin infections to severe illnesses such as septicaemia, toxic shock, endocarditis, and pneumonia.^1^ First described in 1961, the increasing incidence of meticillin-resistant *S aureus* (MRSA), and its spread in hospitals and the community, has posed a major challenge for infectious disease medicine. The evolution of meticillin resistance in *S aureus* is, in part, conferred by the acquisition of one of several staphylococcal cassette chromosome *mec* elements (SCC*mec*),^2^ which carry a gene (*mecA*) that encodes a penicillin binding protein (PBP2a) with low affinity for β-lactam antibiotics.^3^

Most isolates of MRSA in the UK are identified with antimicrobial susceptibility testing by measuring zones of growth inhibition around antibiotic-impregnated discs on agar plates or measurement of minimum inhibitory concentrations (MIC), as recommended by the British Society for Antimicrobial Chemotherapy guidelines.^4^ Meticillin resistance breakpoints are zone diameters of 14 mm or less around 1 μg oxacillin-impregnated discs, zone diameters of 21 mm or less around 10 μg cefoxitin-impregnated discs, an oxacillin MIC of more than 2 mg/L, or a cefoxitin MIC of more than 4 mg/L. Detection of the *mecA* gene by PCR or the detection of PBP2a in a slide agglutination assay^5^ can be used to confirm a diagnosis of MRSA when antimicrobial susceptibility results are borderline.

Before 2003, most MRSA identified belonged to multilocus sequence type clonal complexes (CC) associated with human carriage and infection. The emergence of MRSA CC398 (known as livestock-associated MRSA) in farm animals and human beings has shown that some *S aureus* lineages might not be strongly host-species restricted.^6^ A survey of slaughter pigs in the Netherlands^7^ showed that 39% harboured MRSA sequence type (ST)398 and another survey^8^ showed that 27% of people working at, or living on, a livestock farm in the Netherlands carried livestock-associated MRSA. MRSA ST398 can cause infection in people, with close animal contact being the main risk factor,^9^ suggesting that farm animals could provide a reservoir of MRSA.

Here we report MRSA strains obtained from cattle and human beings, which carry a new SCC*mec* that is undetectable by molecular diagnostic tests used for identification of MRSA.

## Methods

### Bacterial isolates

*S aureus* LGA251 and *S aureus* LGA254 were isolated from a bulk milk sample from a farm in southwest England in May, 2007.^10^ 24 MRSA were obtained from a collection of 940 *S aureus* isolates, submitted from 465 different herds, after antibiotic susceptibility testing of all milk samples from cows with mastitis. The samples had been submitted to one of 14 regional Veterinary Laboratories Agency centres between April, 2006, and September, 2007, for bacteriological characterisation.^11^ None of these isolates tested positive after standard PCR tests for *mecA*.^11^ MRSA isolated from human beings, as detailed in the webappendix (p 11), were provided by the Health Protection Agency (HPA; Addenbrooke's Hospital, Cambridge, UK); the Staphylococcal Reference and Antibiotic Resistance Monitoring Laboratories, HPA (Colindale, London, UK); the Scottish MRSA Reference Laboratory (Glasgow, UK); and the National MRSA Reference Laboratory, Statens Serum Institut (Copenhagen, Denmark). Every centre identified likely candidate isolates for PCR testing for the *mecA* homologue, as detailed in the webappendix (p 11). Submission of all Danish MRSA to the Statens Serum Institut has been mandatory since November, 2006, and all MRSA and meticillin-susceptible *S aureus* submitted since 2007 have been *spa* typed; 678 isolates in 2007, 857 in 2008, 817 in 2009, and 1090 in 2010 were MRSA.

### Procedures

Species identification of selected isolates was confirmed by PCR with primers based on the 16S–23S rRNA spacer region for *S aureus*.^12^ Testing of strains for the presence of PBP2a was done with the Mastalex test (Mast Group, Bootle, UK), which is a slide agglutination assay that detects PBP2a in MRSA by use of latex sensitised with a monoclonal antibody directed against PBP2a.^5^ Molecular detection of *mecA, femB*, and the SCC*mec–orfX* junction was done with PCR, as described previously.^13^, ^14^, ^15^, ^16^, ^17^, ^18^ A comparison of the primer sequences used to test for *mecA* and the target sequences in *mecA* and *mecA*_LGA251_ are shown in the webappendix (p 2). Isolates were genotyped for multilocus sequence type and *spa* type, as described previously.^19^, ^20^

For all test isolates, the MIC of oxacillin and cefoxitin were measured by either Etest (AB Biodisk, Solna, Sweden) or agar dilution,^21^ depending on which laboratory did the test. The disc diffusion technique was used to establish susceptibility of LGA251 and LGA254 to penicillin, oxacillin, cefoxitin, gentamicin, neomycin, ciprofloxacin, tetracycline, erythromycin, clindamycin, fusidic acid, chloramphenicol, teicoplanin, rifampicin, trimethoprim, linezolid, and mupirocin.^22^ To establish whether β-lactam resistance was a result of hyperproduction of β-lactamase, tests were done with and without adjacent discs impregnated with both amoxicillin and clavulanic acid.^22^ The *S aureus* NCTC 12493 strain was used as a control for MRSA and the *S aureus* NCTC 6571 strain was used as a control for meticillin-susceptible *S aureus* (both strains from the National Collection of Type Cultures, HPA, Salisbury, UK). Growth on chromogenic MRSA screening agar, MRSA ID (bioMérieux, Basingstoke, UK), was measured by standard plating and incubation for 18 h at 35°C.

We developed a PCR assay to amplify a region of *mecA* and the novel homologue described here, *mecA*_LGA251_. Primers were based on conserved regions of the *mecA* sequences of previously described *S aureus* strains,^23^ *S aureus* LGA251, and other *Staphylococcus* species (*Staphylococcus epidermidis, Staphylococcus sciuri, Staphylococcus vitulinus, Staphylococcus capitis, Staphylococcus kloosii, and Staphylococcus pseudintermedius*). All the *mecA* sequences were aligned with Bioedit (Ibis Therapeutics, Carlsbad, USA). Primers were chosen from conserved regions with a GC proportion of 40%. The chosen sequences were checked with Primer3 (version 1.1.4) for melting temperatures and self-complementarity,^24^ and pDraw32 (version 1.1.101) was used to confirm the amplicon size and melting temperatures. Primers were as follows: Fw, 5′ TCACCAGGTTCAAC[Y]CAAAA 3′; and Rv, 5′ CCTGAATC[W]GCTAATAATATTTC 3′. Primers for the amplification of the *femB* control gene were obtained from a previously described protocol.^13^ A 25 μL PCR reaction contained final concentrations of 2·5 units of Taq DNA polymerase (Qiagen, Crawley, UK); 1xQ solution (Qiagen); 1xQiagen CoralLoad PCR buffer (Tris-Cl, KCl, [NH_4_]_2_SO_4_, 15 mmol/L MgCl_2_, gel-loading reagent, orange dye, and red dye; pH 8·7; Qiagen); 4 mmol/L of MgCl_2_; 0·8 mmol/L of each dNTP (GeneAmp, Applied Biosystems, Warrington, UK); 0·4 μmol/L of each primer (Operon, Cologne, Germany); and 50 ng of DNA template. A negative control, with no target DNA, was included in the PCR run in the GeneAmp PCR System 9700 (Applied Biosystems). The amplification programme consisted of an initial denaturation step at 94°C for 5 min; 30 cycles of denaturing at 94°C for 1 min, annealing at 55°C for 1 min and extension at 72°C for 2 min; and a final extension at 72°C for 5 min. PCR products were analysed by electrophoresis on a 1% agarose gel, previously stained with ethidium bromide at 0·14 μg/mL (Sigma, Gillingham, UK), and run at 5 V/cm for 45 min. The molecular marker used was a 100 bp ladder (Promega, Southampton, UK). The sizes of the PCR products sequenced after PCR were 356 bp for the *mecA* gene, and 651 bp for the *femB* gene.

We designed a duplex-PCR to detect the *mecA* regulatory genes (primers: *mecIF*, 5′ GACACGTGAAGGCTATGATATAT 3′; *mecIR*, 5′ ATTCTTCAATATCATCTTCGGAC 3′; *mecR1F*, 5′ GGCTCAGTTAAATCATAAAGTTTG 3′; *mecR1R*, 5′ AAATTGCCTTACCATAGCTTGTGT 3′), a duplex-PCR to identify the two cassette recombinase genes (primers: *ccrAF*, 5′ GCAATAGGTTATCTACGTCAAAG 3′; *ccrAR*, 5′ TCTAATGATTGTGCGTTGATTCC 3′; *ccrBF*, 5′ TTCGTGTATCGACAGAAATGCAG 3′; *ccrBR*, 5′ CATCTTTACGAATATCAATACGG 3′), and a single target PCR to amplify the β-lactamase gene *blaZ* (primers: *blaZF*, 5′ AGTCGTGTTAGCGTTGATATTAA 3′; *blaZR*, 5′ CAATTTCAGCAACCTCACTTACTA 3′). The sizes of the expected PCR products were 344 bp for *mecI*, 710 bp for *mecR1*, 932 bp for *ccrA*, 1449 bp for *ccrB*, and 809 bp for *blaZ*. Except for use of an annealing temperature of 58°C instead of 55°C, we used the same method as for the other PCR assay described above.

The whole genome of the *S aureus* isolate LGA251 was sequenced with both capillary sequencing (on ABI 3730xl analysers; Applied Biosystems) and pyrosequencing (on 454 instruments; Life Sciences, Roche Diagnostics Corporation, Branford, CT, USA). A total of 29 300 high quality capillary reads were produced mostly from two subclone libraries (a 2–3 kb insert library and a 3–4 kb library, both with the vector pOTW12). The average read length was 650 bp and these reads represented 6·8 times coverage. The 454 sequencing produced 59·07 Mb data in reads with an average length of 225 bp. The assembly of these reads with Newbler 1.1.03.24 gave 81 contigs greater than 500 bp with a combined length of 2 699 627 bp in six scaffolds.

A combined assembly of the capillary reads, with Phrap (Version 17.0), and the consensus sequences from the 454 assembly (which were converted into overlapping 500 bp sequences) produced 26 contigs (overlapping sequences or clones from which a sequence can be obtained) greater than 2 kb with an N50 of 532 kb. A further 2310 high quality reads were produced to close gaps and to improve the quality of the sequence to finished standard. The sequence was finished and annotated as described previously.^25^ The sequences and annotations of the *S aureus* strain LGA251 genome have been entered in the EMBL database (accession numbers FR821779 for the chromosome and FR821780 for the plasmid).

### Statistical analysis

Temporal trends for annual incidence of detection of *mecA*_LGA251_ in Denmark for the years 2007–10, with all MRSA as the denominator, were assessed with the Cochran-Armitage trend test. Statistical analysis was done with StatXact version 9.

### Role of the funding sources

The sponsors of the study had no role in study design, data collection, data analysis, data interpretation, or writing of the report. The corresponding author had full access to all the data in the study and had final responsibility for the decision to submit for publication.

## Results

LGA251 was resistant to penicillin, oxacillin, and cefoxitin, but susceptible to gentamicin, neomycin, ciprofloxacin, tetracycline, erythromycin, clindamycin, fusidic acid, chloramphenicol, teicoplanin, rifampicin, trimethoprim, linezolid, and mupirocin. LGA251 gave negative reactions in the latex agglutination test for PBP2a, a PCR assay with primers for *mecA*,^13^ and a PCR that amplifies a region of the SCC*mec* (SCC*mec-orfX*).^18^ Culture of LGA251 on agar plates with and without adjacent discs impregnated with both amoxicillin and clavulanic acid indicated that resistance was not mediated by β-lactamase hyperproduction (webappendix p 4). LGA254 had cefoxitin MICs of 24 mg/L and oxacillin Etest (AB Biodisk NA, Culver City, CA, USA) MICs of 12 mg/L, and although resistant to penicillin, was susceptible to gentamicin, neomycin, ciprofloxacin, tetracycline, erythromycin, clindamycin, fusidic acid, chloramphenicol, teicoplanin, rifampicin, trimethoprim, linezolid, and mupirocin. Both LGA251 and LGA254 were sequence type 425 by multilocus sequence type but the *spa* type of LGA251 was t6300 (*spa* repeat: 14-44-12-17-23-18-110-17-17-23-24) and the *spa* type of LGA254 was t6292 (*spa* repeat: 14-44-12-17-23-18-110-17-17-17-23-24).

**Figure 1:**
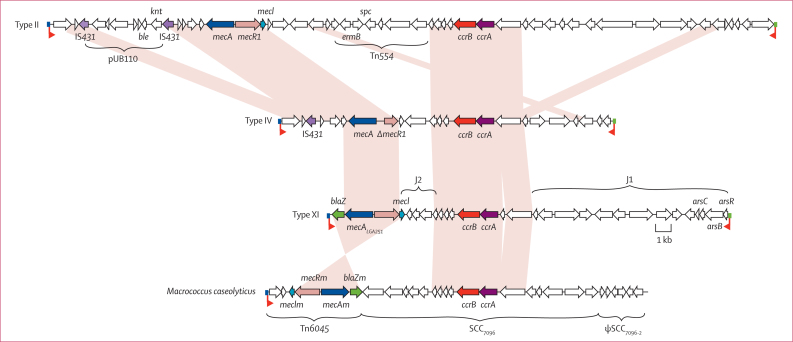
Comparison of the type XI SCC*mec* of LGA251 with other SCC elements Schematic diagram of type II SCC*mec* of meticillin-resistant *Staphylococcus aureus* MRSA252 (top), type IV SCC*mec* of MW2 (upper middle), type XI SCC*mec* of LGA251 (lower middle), and the SCC*mec*-like element of *Macrococcus caseolyticus* JCSC7096 (bottom). The names of the coding sequences associated with drug and metal resistance are provided. Coding sequences are marked in the direction of transcription as arrows. Coding sequences belonging to the *mec* and *ccr* complexes, and IS*431* are coloured, with homologues given the same colour; the individual genes belonging to these groups are named. Light pink shading joins regions that are conserved between elements. The coloured boxes at the end of the SCC elements mark the integration site sequence (blue, *attL*; green *attR*).

The genetic basis of the meticillin resistance in LGA251 was investigated by whole-genome sequencing. Comparative analysis of the 2·8 Mb genome revealed a 29·4 kb SCC*mec* element inserted in the chromosome at the same locus (3′ region of *orfX*) as other SCC*mec* elements, flanked by sequences that match the SCC integration site sequence.^26^ The element contained 29 coding sequences, including homologues of the site-specific recombinase genes *ccrA* and *ccrB*, and the *mecA* gene encoding the penicillin-binding protein PBP2a (figure 1).

The site-specific recombinases CcrA and CcrB were divergent by comparison with those encoded by other SCC*mec* elements; CcrA was classified as *ccrA1* group and CcrB was classified as *ccrB3* group (webappendix p 13). The combination of *ccrA1* and *ccrB3* is novel and has been designated as type-8 *ccr*.

Comparison of the PBP2a encoded by the LGA251 *mecA* (MecA_LGA251_) with proteins in the public sequence databases (Universal Protein Resource; UniProt) indicated that it is divergent in comparison with all other MecA homologues; MecA_LGA251_ was most similar to MecA from the type III SCC*mec* (accession number Q93IC2), having 63% identity at the aminoacid level (70% at the DNA level; webappendix p 13). Phylogenetic analysis of MecA_LGA251_ showed that it belongs to the PBP2a family (data not shown) and is found on a branch outside the clade that includes *S aureus* homologues and homologues from other *Staphylococcus* species (webappendix p 6).

The *mecA*_LGA251_ gene was part of a complex (*mecI–mecR1–mecA–blaZ*) that has a different organisation to other SCC*mec* elements, and has been designated as class E *mec* gene complex. A complex with the same organisation has been identified in a plasmid,^27^ and an SCC*mec*-like element carried by *Macrococcus caseolyticus*,^28^ although the sequence conservation of the two complexes is low (<60% identity at the DNA level).

The LGA251 SCC*mec* element was novel and holds only two of the three joining regions normally seen (J1 and J2; figure 1). The *blaZ* gene was located at the left hand end of the element in a J3 region, but has been designated as part of the class E *mec* gene complex. The J2 region contained genes encoding hypothetical, exported, and membrane proteins, and the J1 region contained hypothetical proteins, an exported protein, membrane proteins, a lipase gene fragment, ABC transporter proteins, and genes associated with arsenic resistance. The sequence was submitted to the International Working Group on the Classification of SCC^29^ and has been given the designation type XI SCC*mec*.

**Figure 2:**
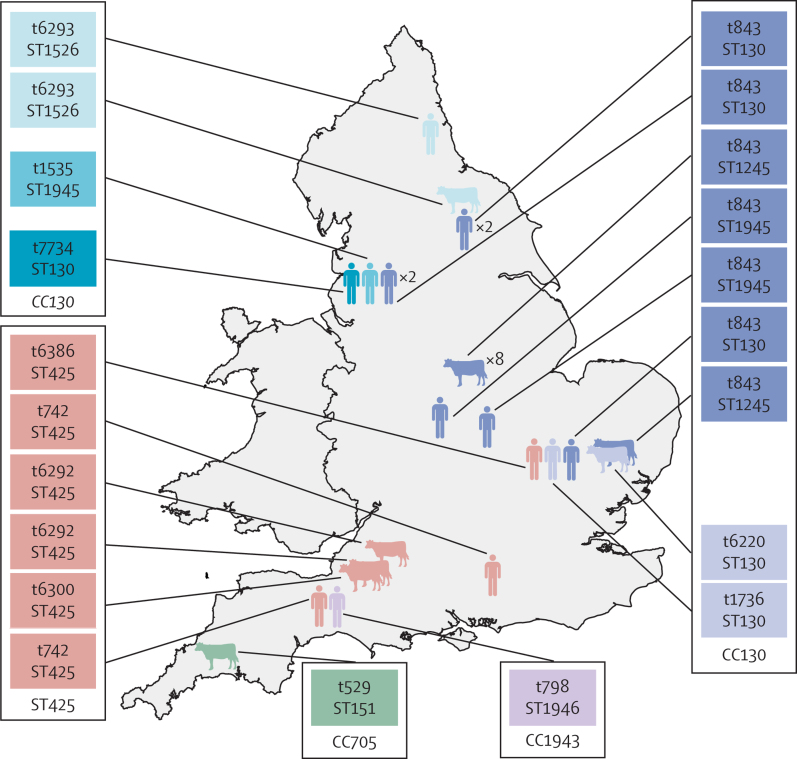
Geographical distribution of the bovine and human meticillin-resistant *Staphylococcus aureus* strains carrying the *mecA*_LGA251_ gene in England The colouring of the symbols and labels indicates common lineage, defined on the basis of *spa* typing, multilocus sequence typing, or both. *spa* types and multilocus sequence types are indicated in the labels.

Details of the geographical distribution, year of isolation, and test results for the bovine isolates are included in the table and figure 2. More than half (13 of 24; 54%) of the MRSA isolates from the survey done by the Veterinary Laboratories Agency were positive by PCR for the *mecA*_LGA251_ gene and for the *mecI, mecR1, ccrA, ccrB*, and *blaZ* genes of type XI SCC*mec*. Sequencing of the *mecA* PCR amplicons from the 13 positive *S aureus* showed that these isolates had an identical sequence to that of *mecA*_LGA251_. The 13 *mecA*_LGA251_-positive *S aureus* isolates were genotyped by multilocus sequence type and *spa* type. One strain was the same sequence type as LGA251 (ST 425), the remainder resolved into four different sequence types.

**Table:** Data for all bovine and human meticillin-resistant *Staphylococcus aureus* strains with the *mecA*_LGA251_ gene, by isolate

	**Host**	**Location**	**Sample**	**Year**	**Multilocus sequence type**	**Clonal complex**	***spa* type**	**Oxacillin MIC (mg/L)**	**Cefoxitin MIC (mg/L)**
LGA251	Bovine	Somerset, England	Bulk milk	2007	425	425	t6300	16	32
LGA254	Bovine	Somerset, England	Bulk milk	2007	425	425	t6292	12	24
C02 467	Bovine	Bury St Edmunds, England	Milk	2006–07	130	130	t6220	32	12
C02 937	Bovine	Langford, England	Milk	2006–07	425	425	t6292	16	12
C03 125	Bovine	Bury St Edmunds, England	Milk	2006–07	1245	130	t843	16	32
C03 362	Bovine	Sutton Bonington, England	Milk	2006–07	1245	130	t843	2	12
C03 363	Bovine	Sutton Bonington, England	Milk	2006–07	1245	130	t843	16	24
C03 364	Bovine	Sutton Bonington, England	Milk	2006–07	1245	130	t843	16	12
C03 365	Bovine	Sutton Bonington, England	Milk	2006–07	1245	130	t843	16	24
C03 366	Bovine	Sutton Bonington, England	Milk	2006–07	1245	130	t843	16	12
C03 367	Bovine	Sutton Bonington, England	Milk	2006–07	1245	130	t843	16	12
C03 370	Bovine	Sutton Bonington, England	Milk	2006–07	1245	130	t843	16	32
C03 371	Bovine	Sutton Bonington, England	Milk	2006–07	1245	130	t843	16	24
C04 288	Bovine	Thirsk, England	Milk	2006–07	1526	130	t6293	8	32
C04 831	Bovine	Truro, England	Milk	2006–07	151	705	t529	16	4
SA227	Human	Cambridge, England	Unknown	2008	130	130	t1736	16	32
02.5099.D	Human	Tayside, Scotland	Finger	2002	1944	130	t843	24	32
08.6601.A	Human	Lothian, Scotland	Nose	2008	1245	130	t1535	32	24
09.2364.C	Human	Ayrshire and Arran, Scotland	Nose	2009	1245	130	t7947	24	32
09.3741.Aa	Human	Western Isles, Scotland	Wound	2009	130	130	t843	8	16
09.4633.Ca	Human	Highland, Scotland	Nose	2009	1764	130	t7485	32	16
09.4657.D	Human	Grampian, Scotland	Wound	2009	1764	130	t7485	16	24
09.7342.H	Human	Grampian, Scotland	Blood	2009	130	130	t843	16	24
09.8549.Q	Human	Highland, Scotland	Wound	2009	1764	130	t7485	32	32
10.1799.W	Human	Tayside, Scotland	Screen	2010	1245	130	t7946	16	16
10.3514.K	Human	Greater Glasgow and Clyde, Scotland	PEG site	2010	1943	1943	t7945	32	48
10.4264.V	Human	Ayrshire and Arran, Scotland	Toe	2010	130	130	t843	32	32
10.7365.L	Human	Greater Glasgow and Clyde, Scotland	Hand	2010	130	130	t843	16	16
H052960441	Human	East England	Not known	2005	130	130	t843	16	64
H061860351	Human	Northwest England	Sputum	2006	130	130	t843	16	16
H074300388	Human	Northwest of england	Skin swab	2007	130	130	t843	16	64
H091100226	Human	East England	Skin swab	2009	425	425	t6386	16	32
H092840495	Human	Northeast England	Nose screen	2009	1526	130	t6293	8	8
H093880936	Human	Southeast England	Ear swab	2009	425	425	t742	16	64
H101740629	Human	Northwest England	Skin swab	2010	130	130	t7734	16	16
H102840444	Human	Southwest England	Nose screen	2010	425	425	t742	8	32
H103360314	Human	Northwest England	Nose screen	2010	1945	130	t1535	16	16
H104700070	Human	Yorkshire and Humber, England	Nose screen	2010	130	130	t843	16	4
H104720074	Human	Yorkshire and Humber, England	Nose screen	2010	130	130	t843	16	4
H105020135	Human	East Midlands, England	Nose screen	2010	1945	130	t843	8	4
H105060288	Human	West Midlands, England	Nose screen	2010	1945	130	t843	8	4
H105140339	Human	Southwest England	Nose screen	2010	1946	1943	t978	16	4
43066	Human	Næstved, Denmark	Wound	2004	130	130	t843	2	16
43067	Human	Næstved, Denmark	Wound	2004	130	130	t843	16	32
45454	Human	Herlev, Denmark	Skin	2005	130	130	t843	6	16
47034	Human	Herlev, Denmark	Unknown	2005	130	130	t843	6	32
61425	Human	Viborg, Denmark	Blood	2008	130	130	t843	3	12
65727	Human	Sønderborg, Denmark	Nose/mouth	2009	n/d	*130*	t843	16	64
66519	Human	SSI, Denmark	SSTI	2009	n/d	*130*	t843	8	8
66712	Human	Aalborg, Denmark	SSTI	2009	130	130	t843	4	16
67909	Human	Vejle, Denmark	SSTI	2009	n/d	130	t843	0·75	8
70355	Human	Hillerød, Denmark	Nose/mouth	2010	130	130	t843	4	24
70782	Human	Slagelse, Denmark	Eye/ear	2010	n/d	130	t843	6	32
70956	Human	Slagelse, Denmark	SSTI	2010	n/d	130	t843	4	48
71274	Human	Slagelse, Denmark	Blood	2010	n/d	130	t843	12	32
71795	Human	Slagelse, Denmark	SSTI	2010	n/d	130	t843	1·5	8
71950	Human	Aalborg, Denmark	Eye/ear	2010	n/d	130	t843	16	64
71957	Human	Slagelse, Denmark	Unknown	2010	n/d	130	t843	1·5	16
72270	Human	Nykbøbing F, Denmark	Screening	2010	n/d	130	t843	6	24
72890	Human	Aalborg, Denmark	SSTI	2010	n/d	130	t843	24	48
72976	Human	Vejle, Denmark	Fluid	2010	n/d	130	t843	4	16
73532	Human	Herning, Denmark	SSTI	2010	n/d	130	t843	4	16
73760	Human	Hillerød, Denmark	Blood	2010	n/d	130	t843	16	64
73829	Human	Vejle, Denmark	Unknown	2010	n/d	130	t843	8	16
75022	Human	Slagelse, Denmark	Unknown	2011	n/d	130	t1535	12	32
Enr.7594/75	Human	SSI, Denmark	Blood	1975	n/d	130	t843	3	16

PEG=percutaneous endoscopic gastrostomy. SSI=Statens Serum Institut. SSTI=skin and soft tissue infection. MIC=minimum inhibitory concentration. n/d=not done (clonal complexes for these isolates are predictions based on *spa* type).

Searches for human MRSA isolates yielded 51 isolates that tested positive by PCR for *mecA*_LGA251_ from likely candidates of about 120 000 clinical isolates: the only one tested from Cambridge, 14 of 25 tested from London, 12 of 16 tested from Glasgow, and 24 of 32 tested from Copenhagen. The results of tests are listed in the table and the geographical distribution of the *mecA*_LGA251_ isolates is shown in figure 2 for England and in the webappendix for Scotland (p 7) and Denmark (p 8). The oxacillin MICs ranged from 0·75 mg/L to 32 mg/L, with an MIC_50_ of 4 mg/L and MIC_90_ of 16 mg/L. The cefoxitin MICs ranged from 4 mg/L to 64 mg/L, with an MIC_50_ of 8 mg/L and MIC_90_ of 24 mg/L. All the British isolates were multilocus sequence typed together with seven of 24 Danish isolates. In the Danish isolates *mecA*_LGA251_ was detected in none of 678 isolates in 2007, one of 857 isolates in 2008 (0·12%, 95% CI 0–0·35%), four of 817 isolates in 2009 (0·49%, 0·01–0·97), and 13 of 1090 isolates (0·67%, 0·61–1·95) in 2010 (trend for increase: p=0·0002).

The evolutionary relation of the MRSA sequence types that carry *mecA*_LGA251_ was compared within this population and with the *S aureus* population as a whole, considering the most frequently reported sequence types (ten or more isolates reported per sequence type).^30^ Analysis of the population framework shows that *mecA*_LGA251_ homologues are present in phylogenetically distinct *S aureus*, indicating that multiple independent acquisitions of the gene have occurred (webappendix p 9).

## Discussion

A novel *mecA* homologue, *mecA*_LGA251_, associated with resistance to β-lactam antibiotics was present in clinical MRSA isolates from the UK and Denmark, and bovine milk samples from the UK. Our search for *mecA*_LGA251_ MRSA has been limited to existing collections comprising isolates collected for various reasons and for which incomplete clinical data or strain typing were available. Therefore, interpretations made from our results are tentative and need to be confirmed by more systematic studies with appropriate methodology.

The detection of this gene in MRSA isolates cultured from blood and infected wound sites is strong circumstantial evidence that these organisms are capable of causing clinical disease. Whether disease caused by *mecA*_LGA251_ *S aureus* is identical to that caused by conventional MRSA is not clear without evidence from formal epidemiological and virulence studies. Isolate collections were systematically searched for isolates that seemed to be β-lactam resistant, but tested negative by PCR for *mecA* or by slide agglutination test for PBP2a, and from a search for MRSA isolates that had *spa* types associated with *mecA*_LGA251_ MRSA (webappendix p 11). The Danish isolate archive is a complete national collection. These results reveal that detection rates of *mecA*_LGA251_ MRSA increased substantially between 2007 and 2010. These detection rates are good incidence estimates for those years in Denmark because the archive for 2007–10 contains all Danish MRSA, and because all MRSA isolates were *spa*-typed.

In this study, we provide evidence of an association between SCC*mec*_LGA251_ and β-lactam resistance—which includes meticillin resistance—in *S aureus*. Genetic manipulation of *S aureus* strains to show that the insertion or removal of the *mecA* homologue leads to acquisition or loss of β-lactam resistance will need to be done to show direct linkage, and such studies are underway. However, we were able to search the entire genome of LGA251 for evidence of other genes that might confer resistance. Although a β-lactamase gene (*blaZ*) is present in SCC*mec*_LGA251_ the inability of clavulanate (a β-lactamase inhibitor) to ablate resistance indicates that resistance is unlikely to be caused by β-lactamase. This finding is consistent with the fact that penicillinase hyperproducers do not show heteroresistance,^31^ as was seen with LGA251 (panel). The expected complement of genes that encode PBP is present (PBP1, PBP2, PBP3, and PBP4) in a highly conserved form compared with other sequences from other *S aureus* isolates (data not shown), and we recorded no additional PBPs. The most likely explanation for the β-lactam resistance seen in LGA251 is that it is mediated by the *mecA* homologue present in the type XI SCC*mec*.

Panel **Research in context**

**Systematic review**

The discovery of a novel *mecA* homologue prompted a search of public sequence databases (UniProt) with the predicted aminoacid sequence for similar *mecA* homologues. No identical sequence was found and the closest match had 63% identity at the aminoacid level. We searched PubMed with the terms “cattle”, “MRSA”, “livestock”, and “MRSA” for any evidence of livestock-associated meticillin-resistant *S aureus* (MRSA) that were not accounted for by known SCC*mec* types. None were found.

**Interpretation**

Advice on the detection and treatment of infections with *mecA*_LGA251_ MRSA by antimicrobial susceptibility testing is no different to the detection and treatment of infections with other MRSA strains. However, clinicians should be aware that molecular techniques of detection of MRSA with PCR or slide agglutination tests do not detect *mecA*_LGA251_ MRSA. This means that when these tests are used either for primary detection, or for confirmation of MRSA, a small chance exists that a false-negative result is obtained. The data presented in this paper suggest that the prevalence of *mecA*_LGA251_ MRSA is likely to be in the range of one in 100 to one in 500 of total MRSA in the UK and Denmark. The discovery of the same *mecA*_LGA251_ MRSA in dairy cows suggests that these animals might provide a reservoir of infection and close links with farms or contact with dairy cattle could be risk factors that increase the likelihood of *mecA*_LGA251_ MRSA carriage or infection in patients. Until better evidence is generated from appropriate observational or experimental studies, this study provides the best evidence to inform clinical decisions concerning this new discovery.

The discovery of this new *mecA* homologue raises issues about the detection and confirmation of MRSA. Irrespective of whether an infection is caused by *mecA*_LGA251_ MRSA or conventional MRSA, after culture and antimicrobial susceptibility testing, appropriate decisions about care of patients can be made. However, when existing PCR or monoclonal antibody methods are used as the only method to detect MRSA, or when these methods are used to confirm provisional detection of MRSA, then *mecA*_LGA251_ *S aureus* will be wrongly diagnosed as meticillin susceptible. The use of new PCR primers such as those described in this paper, and the production of new monoclonal antibodies, would address this problem.

Another important question concerns the potential role of cattle in the epidemiology of human MRSA infections caused by *mecA*_LGA251_ MRSA. Data from the Veterinary Laboratories Agency MRSA survey suggest that *mecA*_LGA251_ could be present in up to 1·4% (95% CI 0·6–2·1%) of *S aureus* bovine mastitis isolates, and is present in up to 2·8 % (1·3–4·3) of herds. The data described in this paper do not provide direct evidence of transmission between cattle and people. However, four pieces of circumstantial evidence suggest that cattle could be the epidemiological source. First, the human isolate strain types were either CC130 (48 of 66)—a lineage that is thought to be unique to animals^32^—or other strain types detected in cattle during this study and not previously reported in human beings. Second, none of the strain types or *spa*-types carrying *mecA*_LGA251_ come from lineages previously associated with human MRSA carriage or infection. Third, in England, where data from both cattle and human beings are available, evidence of geographical association in both human and bovine isolates exists (ie, human and bovine ST425 in the southwest, human and bovine ST130 in the east, and human and bovine ST1526 in the northeast; figure 2). Fourth, the Veterinary Laboratories Agency survey looking for MRSA in dairy cattle noted no other (ie, human) MRSA,^11^ although there are infrequent reports of human-associated lineages of *mecA*-positive MRSA causing mastitis in cattle in other countries.^33^ Such evidence suggests that a bovine reservoir exists, from which *mecA*_LGA251_ MRSA is transmitted to people. Pasteurisation of milk will prevent any risk of infection via the food chain but individuals in close contact with cattle could be at higher risk of carriage. Further research is needed to test this hypothesis.

The absence of diversity between the *mecA*_LGA251_ *S aureus* isolates from Denmark and those from the UK could be a result of a searching bias (ie, that *spa*-type t843 was used as one of the searching criteria, and by testing for known *spa* types only we might have missed *mecA*_LGA251_ MRSA that had different *spa* types). The discovery of an isolate from a sample obtained from a patient in 1975 possessing the same *spa*-type shows that the *mecA* homologue has been in *S aureus* for at least 36 years, and that the Danish lineage could have changed very little during this period. This early Danish isolate also suggests that *mecA*_LGA251_ *S aureus* might have originally been a human strain. The presence of type XI SCC*mec* in four separate multilocus sequence type lineages, and the fact that it is bounded by integration site sequence repeats and has intact site-specific recombination components, suggests that it has the potential to be transferred to other *S aureus* lineages in the future.

The search for *mecA*_LGA251_ MRSA also yielded several isolates that had an MRSA phenotype but no *mecA* gene that could be detected by PCR. These isolates might possess other *mecA* homologues or other mechanisms leading to β-lactam resistance. The discovery of this previously undetected *mecA* homologue is potentially of public health importance. Diagnostic protocols, whether for clinical or epidemiological purposes, should consider the ramifications of not detecting *S aureus* strains that carry this new *mecA* homologue.

## Web Extra Material

Supplementary webappendix
