# Supplementary material for: Meticillin-resistant Staphylococcus aureus with a novel mecA homologue in human and bovine populations in the UK and Denmark: a descriptive study
Source: Lancet Infect Dis. 2011 Aug;11(8):595–603. doi: 10.1016/S1473-3099(11)70126-8 (PMC3829197; doi:10.1016/S1473-3099(11)70126-8)
Supplement: Supplementary webappendix [file mmc1.pdf]

THE LANCET Infectious Diseases

Supplementary webappendix

This webappendix formed part of the original submission and has been peer reviewed. We post it as supplied by the authors.

Supplement to: García-Álvarez L, Holden MTG,Lindsay H, et al. Meticillin-resistant *Staphylococcus aureus* with a novel *mecA* homologue in human and bovine populations in the UK and Denmark: a descriptive study. *Lancet Infect Dis* 2011; published online June 3. DOI:10.1016/S1473-3099(11)70126-8.

Supplementary Figures and Tables

Title

Meticillin-resistant *Staphylococcus aureus* with a novel *mecA* homologue emerging in human and bovine populations in the UK and Denmark: a descriptive study

Authors

Laura García-Álvarez, Matthew. T. G. Holden, Heather Lindsay, Cerian R. Webb, Derek F. J. Brown, Martin D. Curran, Enid Walpole, Karen Brooks, Derek J. Pickard, Christopher Teale, Julian Parkhill, Stephen D. Bentley, Giles F. Edwards, E. Kirsty Girvan, Angela M. Kearns, Bruno Pichon, Robert L.R. Hill, Anders Rhod Larsen, Robert L. Skov, Sharon J. Peacock, Duncan J. Maskell, Mark A. Holmes

```
LGA251            170  AAGAAATTGTAGATAGGAATAAAAAATTTACAAAGATTTA  210     12/25
Perez-Roth 2001        --------GTAGAAATGACTGAACGTCCGATAA--------
MRSA252           170  ATGGTGAAGTAGAAATGACTGAACGTCCGATAAAAATATAT  210     25/25


LGA251           1286  GGCAAAAAGATGCATCATGGGGAATTATAATATCACAAGA  1326    13/21
Poulsen 2003           ----------GGGATCATAGCGTCATTATTC----------
MRSA252           370  AAGTTAGATTGGGATCATAGCGTCATTATTCCAGGAATGCA   410    21/21


LGA251           1032  TACAGCATTACAACCAAAAACTGGAGAAATT  1062             7/21
Kondo 2007             ----TGCTATCCACCCTCAAACAGG------
MRSA252          1040  GTACTGCTATCCACCCTCAAACAGGTGAATT  1070             21/21


LGA251           1171  AAATTTCAAATCACTACATCACCAGGTTCAA  1201             17/22
Oliveira 2002          ----TCCAGATTACAACTTCACCAGG-----
MRSA252          1180  AAGTTCCAGATTACAACTTCACCAGGTTCAA  1210             22/22


LGA251           1611  TAACGGAAATATACAAAATCCTCATGTTTTA  1641             12/20
McDonald 2005          ----GGCAATATTACCGCACCTCA-------
MRSA252          1620  TAATGGCAATATTAACGCACCTCACTTATTA  1650             19/20


LGA251           1181  TCACTACATCACCAGGTTCAACCCAAAAAAT  1211             20/20
Garcia-Alvarez         --------TCACCAGGTTCAACYCAAAA---
MRSA252          1190  TTACAACTTCACCAGGTTCAACTCAAAAAAT  1220             20/20
```

Figure S1: Diagram illustrating the alignment of six different PCR primers with the matching sections of the gene nucleotide sequence of the *mecA* from LGA251 and MRSA252.

All sequences are shown from 5' to 3'. The base pair numbers of the start and end of the sequence of the *mecA* gene are shown to either side of the sequences. The $mecA_{LGA251}$ sequence is shown in the first line of each set of sequences (LGA251) and the *mecA* sequence from MRSA252[1] is shown in the bottom line. Only the forward primer from each primer pair that was tested against LGA251 is shown. The primers tested were from: Perez-Roth 2001,[2] Poulsen 2003,[3] Kondo 2007,[4] Oliveira 2002,[5] McDonald 2005,[6] and Garcia-Alvarez (the primers described in this paper). The first five primers represented (designed to detect the *mecA* gene in MRSA) produced a negative result with $mecA_{LGA251}$. Red text in the nucleotide sequence shows homology of $mecA_{LGA251}$ and the primer sequence with the *mecA* of MRSA252. Green text shows where the primer is designed to recognise two different nucleotides. Blue text indicates a mismatch between the primer and the MRSA252 *mecA* sequence. The numbers in the right hand column show the proportion of nucleotides in the target genome that match the primer sequence.

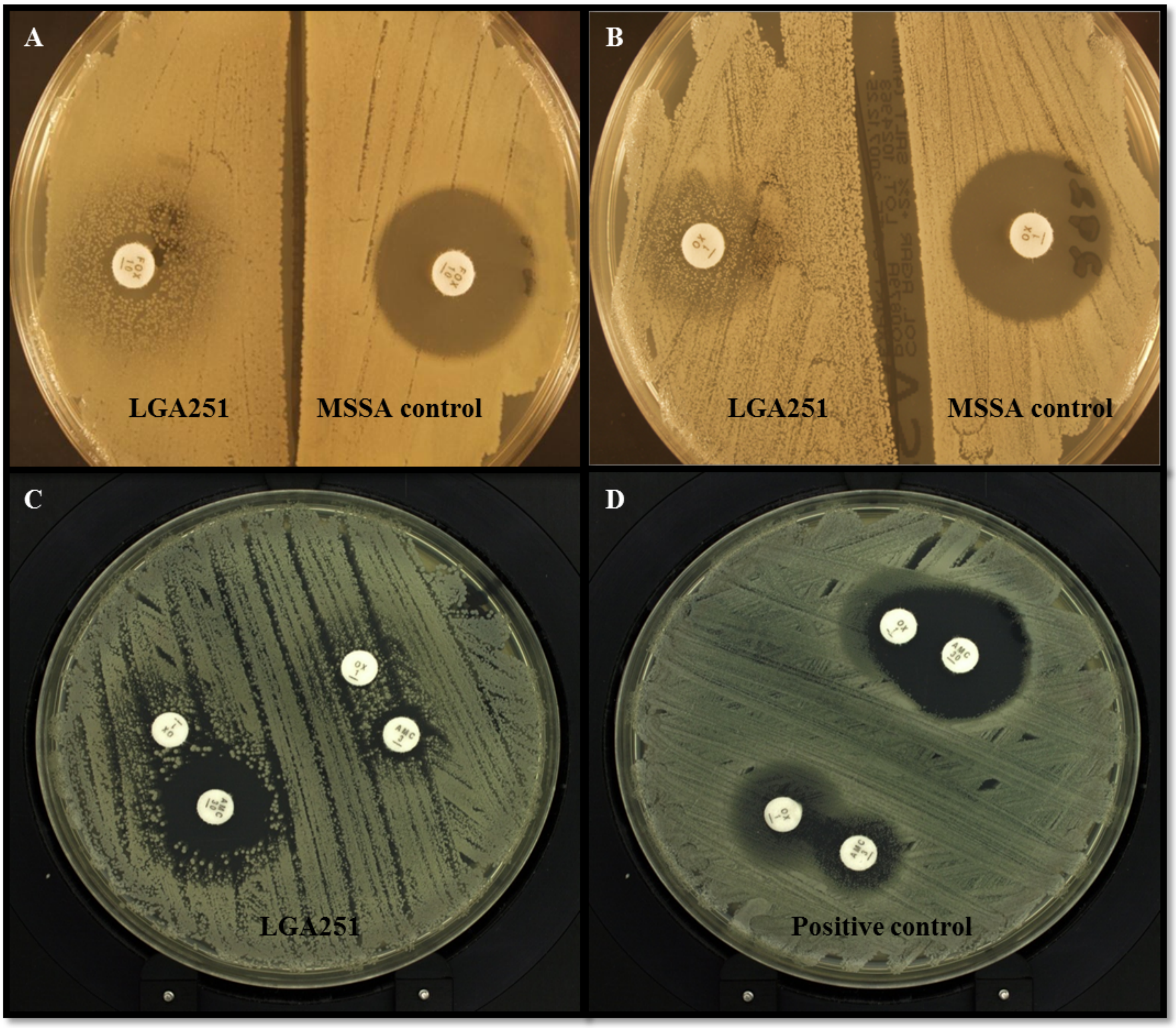
A
LGA251
MSSA control
B
LGA251
MSSA control
C
LGA251
D
Positive control

Figure S2: Antimicrobial phenotypic results for *Staphylococcus aureus* strain LGA251.

The British Society for Antimicrobial Chemotherapy (BSAC) provides guidelines on the methodology of antimicrobial susceptibility testing and the breakpoints that define the MRSA phenotype.[7] Isolates that have zones of inhibition of ≤ 21mm diameter with 10 µg cefoxitin discs, and ≤ 14mm diameter with 1 µg oxacillin discs are considered to be MRSA. Some hyper-producers of β-lactamase give zones within the range of 7-14 mm with 1 µg oxacillin discs (cefoxitin is largely resistant to the action of β-lactamase). Identification of hyper-producers of β-lactamase can be made using amoxicillin-clavulanic acid discs adjacent to a 1 µg oxacillin disc. The clavulanic acid is a β-lactamase inhibitor and the diffusion of clavulanic acid towards the oxacillin disc will create an area between the two discs that is completely devoid of growth if the isolate is a hyper-producer of β-lactamase. The panels in this figure illustrate the result of antimicrobial susceptibility carried out under BSAC guidelines using antimicrobial disc diffusion. (A) Disc diffusion test using 10 µg cefoxitin discs (FOX 10) on Iso-sensitest agar, incubated at 35ºC for 20 hours. MSSA control refers to NCTC 6571 strain; (B) Disc diffusion test using 1 µg oxacillin discs (OX 1) on Columbia agar containing 2% NaCl, incubated at 30ºC for 24 hours. MSSA control refers to NCTC 6571; (C) Oxacillin (1 µg) disc diffusion tests with adjacent amoxicillin-clavulanic acid discs (3 µg AMC 3, and 30 µg AMC 30) on Columbia agar containing 2% NaCl, incubated at 30°C for 24 hours. (D) As C; positive control refers to a penicillinase hyper-producing strain.

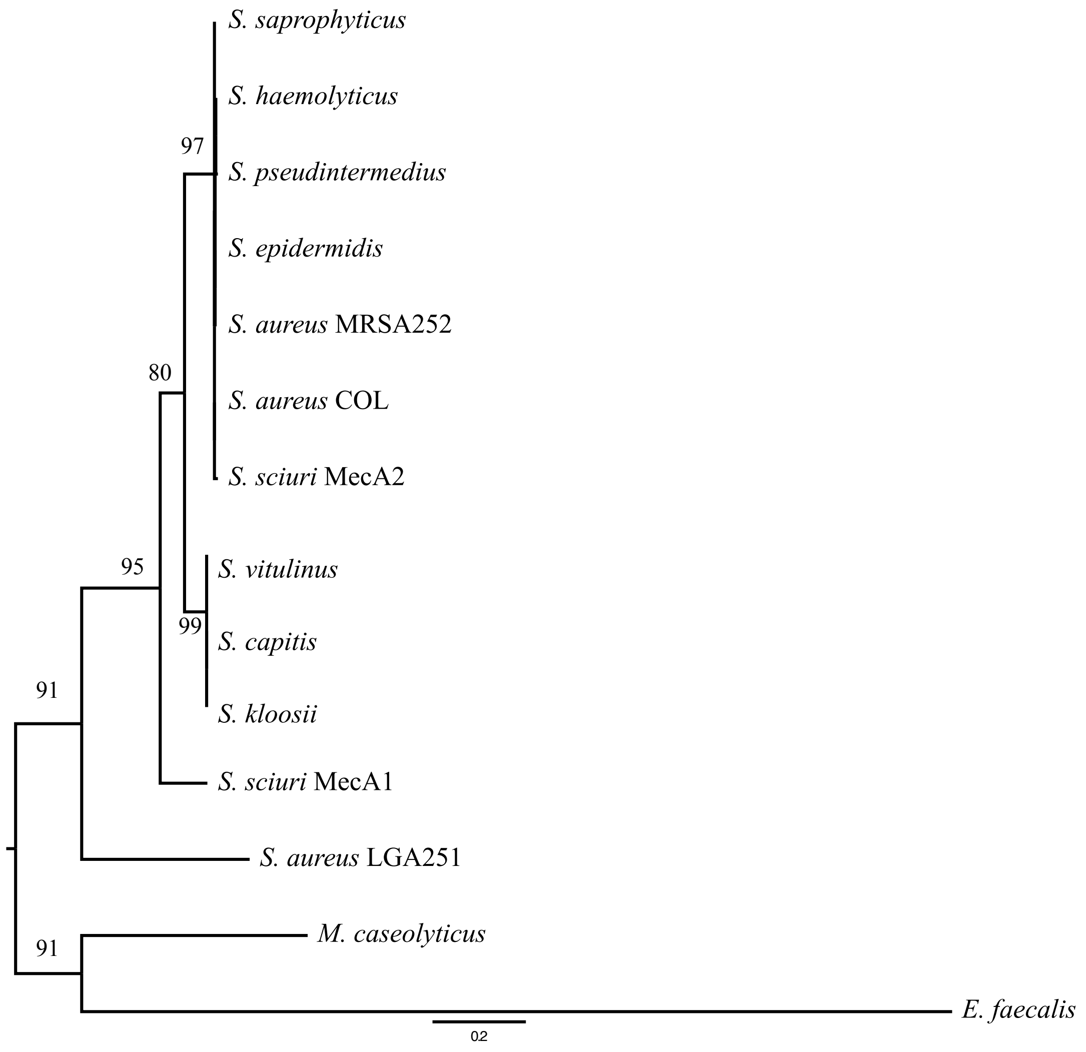

Figure S3: Phylogenetic relationships of MecA homologues.

Maximum likelihood tree constructed with MecA homologues from: *S. aureus* strains LGA251 (SARLGA251_00260), COL (UniProt accession number Q5HJW3), and MRSA252 (Q6GKQ7); *M. caseolyticus* (B9EC93); *S. sciuri* MecA1 (UniProt accession number O54277); *S. sciuri* MecA2 (O54283); *S. pseudintermedius* (B5M0C8); *S. kloosii* (Q4GXY3); *S. capitis* (Q4GXY1); *S. vitulinus* (Q4GXX7); *S. saprophyticus* (B2DCL8); *S. epidermidis* (Q5HK31); *S. haemolyticus* (Q4LAC5); *E. faecalis* (Q6PY40). The numbers at the branches are bootstrap values indicating the support for the branch. The scale bar represents 0.2 amino acid substitutions per site. Sequence alignments and phylogenetic analyses were performed using Seaview (v4.0).[8] The model of sequence evolution used was the WAG model with gamma-distributed rate variation. The tree was drawn with FigTree (v1.2.2; http://tree.bio.ed.ac.uk/software/figtree/)

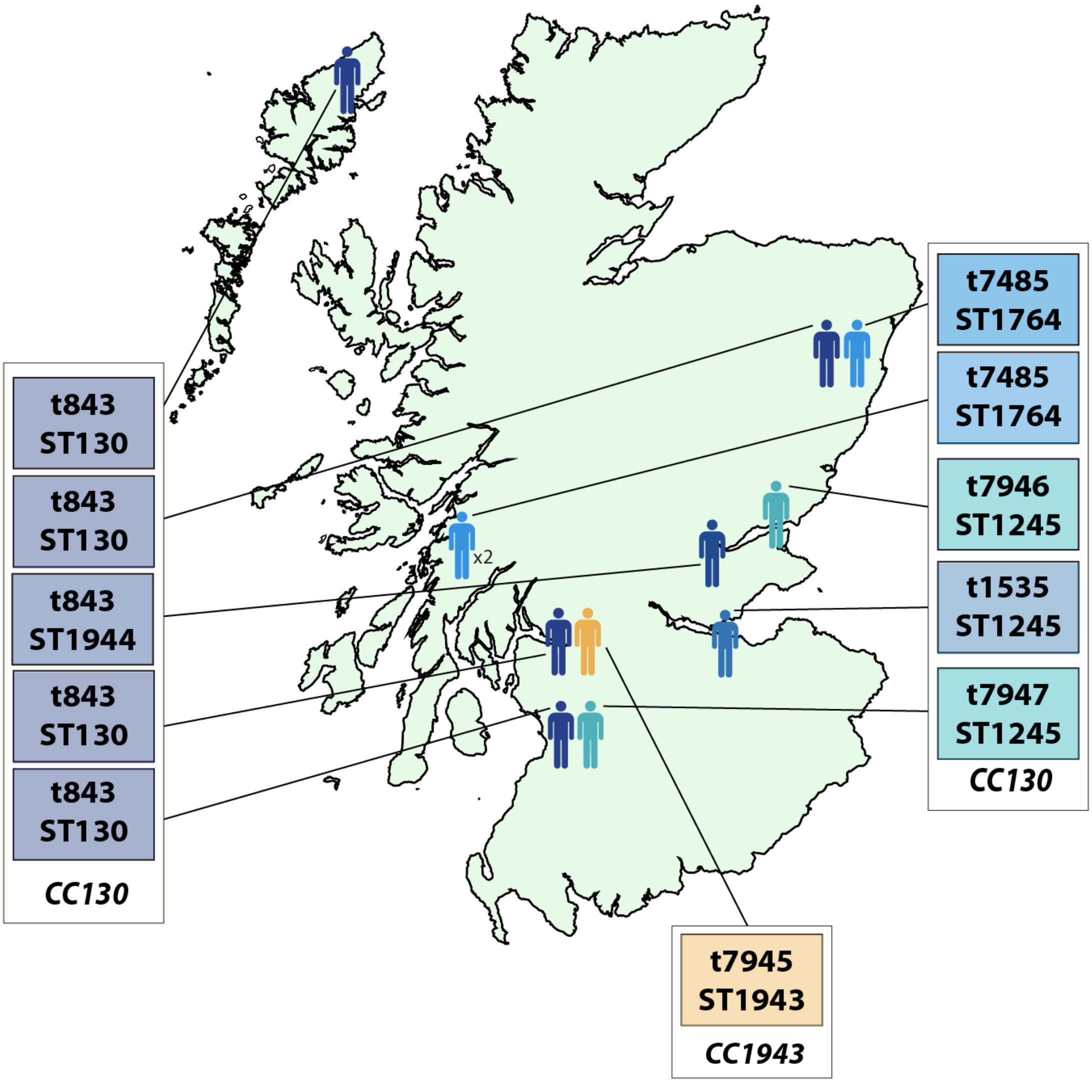

Figure S4: **Geographical distribution of human MRSA strains carrying the novel *mecA* gene in Scotland.** Map of Scotland showing the approximate locations of where the *mecA*$_{LGA251}$ positive isolates were submitted or obtained. The colouring of the symbols and labels indicates common lineage based on *spa* typing and/or multi locus sequence type (MLST) data. The *spa* types together with MLST sequence types (STs) are indicated in the labels.

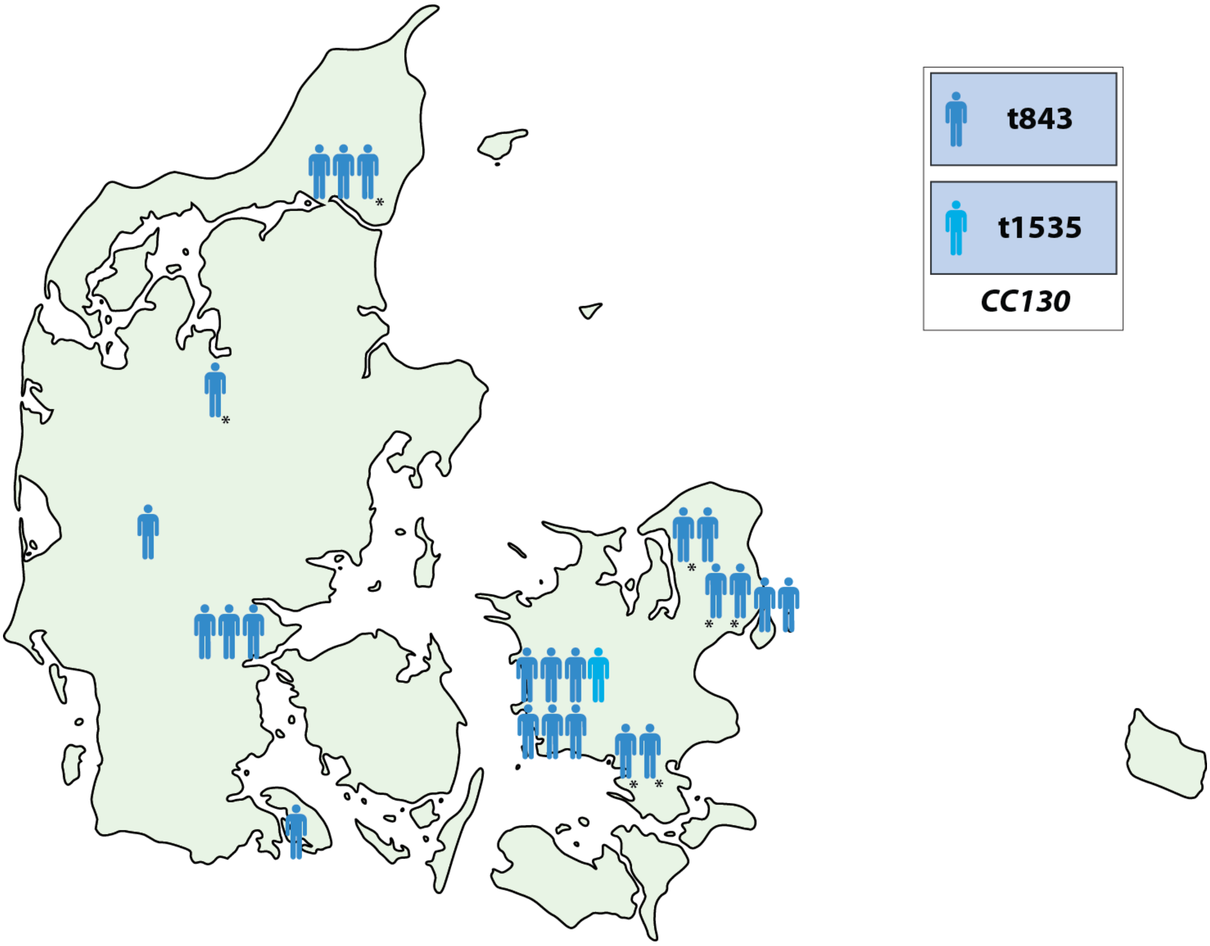

Figure S5: **Geographical distribution of human MRSA strains carrying the novel *mecA* gene in Denmark.** Map of Denmark showing the approximate locations from which the *mecA*$_{\text{LGA251}}$ positive isolates were submitted. The colouring of the symbols and labels indicates common lineage based on *spa* typing and/or multi-locus sequence type (MLST). The *spa* types are indicated in the labels. A sample of seven isolates were subjected to MLST, all were identified as ST130 and are indicated by asterisks (*). All isolates belong to clonal complex (CC) 130 or are predicted to be members of CC130 on the basis of their *spa* type.

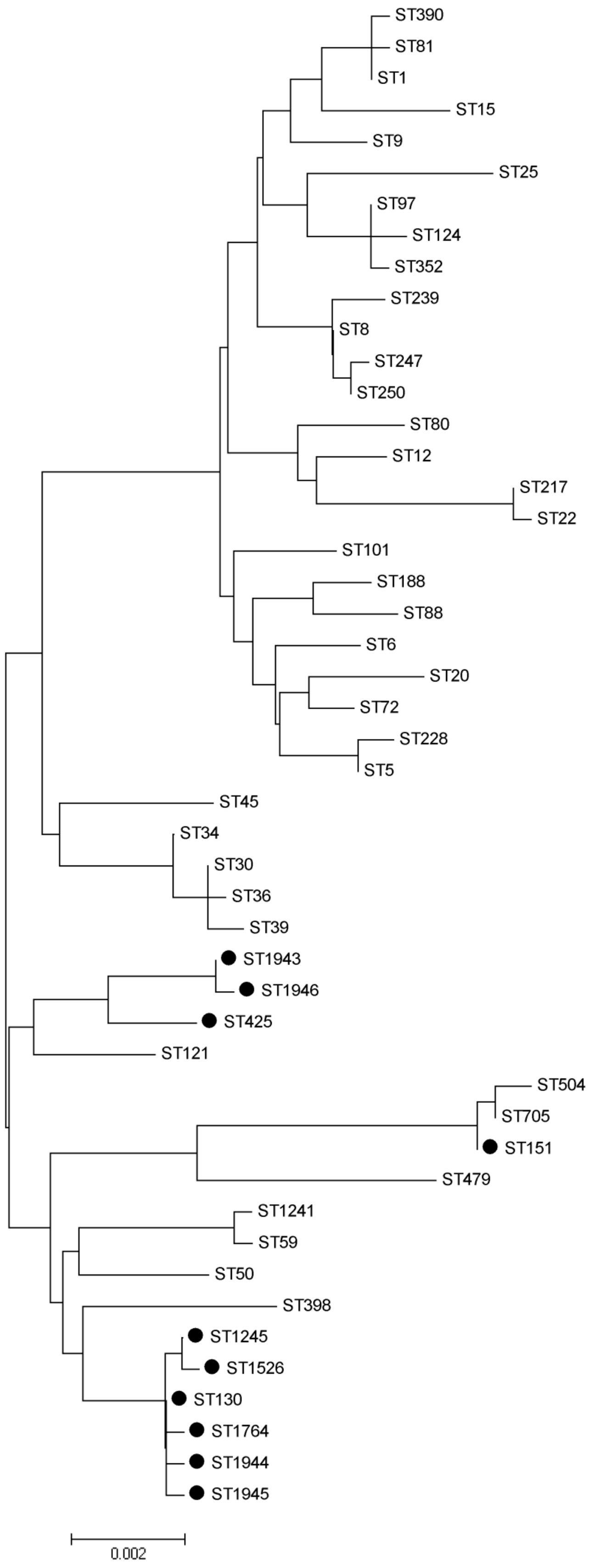
ST390
ST81
ST1
ST15
ST9
ST25
ST97
ST124
ST352
ST239
ST8
ST247
ST250
ST80
ST12
ST217
ST22
ST101
ST188
ST88
ST6
ST20
ST72
ST228
ST5
ST45
ST34
ST30
ST36
ST39
ST1943
ST1946
ST425
ST121
ST504
ST705
ST151
ST479
ST1241
ST59
ST50
ST398
ST1245
ST1526
ST130
ST1764
ST1944
ST1945
0.002

Figure S6: Phylogenetic tree comparing multi-locus sequence types (STs) of *Staphylococcus aureus*. The most frequently reported STs within www.mlst.net[9] (n≥10 isolates reported per ST) and those STs carrying the *mecA*$_{LGA251}$ homologue (indicated by circles) were included. The evolutionary history was inferred using the Neighbor-Joining method. The tree is drawn to scale, with branch lengths in the same units as those of the evolutionary distances used to infer the phylogenetic tree. The evolutionary distances were computed using the Kimura 2-parameter method and are in the units of the number of base substitutions per site. Evolutionary analyses were conducted in MEGA5 (http://www.megasoftware.net/).

Date	Event	Sample collection	Denominator	Selection criteria	Number of isolates tested	Number positive for *mecA*$_{LGA251}$
2007	Screening of bovine *S. aureus* isolates for MRSA	Isolates from bulk and quarter milk samples from 48 herds in England	182	oxacillin and cefoxitin resistant, and then *mecA*-ve	2	2
2008	*Whole genome sequencing of LGA251*					
2009	Screening of 24 *mecA* negative MRSA from VLA	MRSA survey performed in 2006/7 of *S. aureus* from bovine mastitis samples	940 isolates from 465 farms	*mecA* –ve<br>oxacillin and/or cefoxitin resistant	24	13
2009	Screening of human MRSA isolates from Cambridge for *mecA*	500 unique isolates from MRSA testing at Addenbrooke's hospital in 2008	500 MRSA, routine screening and clinical isolates	*mecA* –ve<br>oxacillin and/or cefoxitin resistant	1	1
2010	*Distribution of PCR primers for mecA homologue to national reference labs for S. aureus*					
2010/11	Scotland	*S. aureus* isolates from 1997-2011. Majority of *spa*-typing performed on samples from 2007. All bacteraemias from Scotland are collected. Other isolates are submitted for a variety of reasons.	Approximately 100,000	*spa*-types associated with *mecA* homologue or *mecA* –ve oxacillin and/or cefoxitin resistant	16	12
2011	England	*S. aureus* isolates from 2004-2011. Majority of *spa*-typing performed on samples from 2007. Isolates are submitted for a variety of reasons.	Approximately 10,000	*spa*-types associated with *mecA* homologue or *mecA* –ve with oxacillin MIC >=4mg/L	25	14
2011	Denmark	Some MRSA from 1960's. All MRSA collected from 1988. All MRSA from 2007 are *spa*-typed and some from before this period.	Approximately 10,000 of which 4,264 are *spa*-typed	*spa*-types associated with *mecA* homologue or *mecA* –ve with cefoxitin MIC >=12mg/L	32	24

Table S1: Details of the sequence of events concerning the discovery of *mecA*$_{LGA251}$ gene and the search for isolates possessing SCC*mec*$_{LGA251}$.

A summary of the bacteriological collections searched, the search criteria used, the numbers of isolates matching the search criteria, and the numbers testing positive using PCR primers designed to detect *mecA*$_{LGA251}$ are listed. Abbreviations: *mecA*-ve, *mecA*-negative; VLA, Veterinary Laboratories Agency.

CDS	“Top hits” in UniProt (matches)	Similarity %	Query position	Query length	Match position	Match length
blaZ	β-lactamase in *Staphylococcus aureus*, plasmid [D2J684]	68	1-279	283	15-293	295
mecA	Penicillin-binding protein 2a in:					
	S. aureus [Q93IC2]	63	1-665	665	1-668	668
	S. epidermidis [Q5HK31]	63	1-665	665	1-668	668
	S. sciuri [Q799P0]	63	1-665	665	1-668	668
	S. vitulinus [Q4GXY5]	63	1-665	665	1-668	668
	S. kloosii [Q4GXY4]	63	1-665	665	1-668	668
	S. pseudointermedius [B1GVE2]	63	1-665	665	1-668	668
mecR1	Methicillin-resistance regulatory protein MecR1 in:					
	S. aureus [Q6GKQ6]	44	1-583	584	1-585	585
	S. sciuri [Q799P1]	44	1-583	584	1-585	585
	S. saprophyticus [B2DCL9]	44	1-583	584	1-585	585
	S. pseudointermedius [B1GVE1]	44	1-583	584	1-585	585
mecI	Methicillin-resistance repressor MecI in:					
	S. pseudointermedius [B1GVH4]	67	1-123	124	1-123	123
	S. aureus [Q799K4]	66	1-123	124	1-123	123
	S.epidermidis [Q5HK33]	66	1-123	124	1-123	123
ccrA	Cassette chromosome recombinase A in:					
	S. saprophyticus [B2DCN1]	86	1-449	449	1-449	449
	S. aureus [Q5HJV5]	84	1-448	449	1-448	449
ccrB	Cassette chromosome recombinase B in:					
	S. aureus [Q9RHV7]	92	1-542	542	1-542	542
	S. pseudointermedius [B1GVG2]	92	1-542	542	1-542	542

Table S2: Best matching-proteins for the nucleotide coding sequences (CDS) identified within the novel SCC*mec* type XI.

The “top hits” [accession number] retrieved from the Universal Protein Resource (UniProt) as a result of a BLAST search (protein level), and the similarity percentages between the query sequence and the “top hit” are presented. Information on the alignment length between query and match is also provided.

References:

1. Holden MT, Feil EJ, Lindsay JA, Peacock SJ, Day NP, Enright MC, et al. Complete genomes of two clinical *Staphylococcus aureus* strains: evidence for the rapid evolution of virulence and drug resistance. Proc Natl Acad Sci U S A. 2004; **101**(26): 9786-91.
2. Perez-Roth E, Claverie-Martin F, Villar J, Mendez-Alvarez S. Multiplex PCR for simultaneous identification of *Staphylococcus aureus* and detection of methicillin and mupirocin resistance. Journal of clinical microbiology. 2001; **39**(11): 4037-41.
3. Poulsen AB, Skov R, Pallesen LV. Detection of methicillin resistance in coagulase-negative staphylococci and in staphylococci directly from simulated blood cultures using the EVIGENE MRSA Detection Kit. J Antimicrob Chemother. 2003; **51**(2): 419-21.
4. Kondo Y, Ito T, Ma XX, Watanabe S, Kreiswirth BN, Etienne J, et al. Combination of multiplex PCRs for staphylococcal cassette chromosome *mec* type assignment: rapid identification system for *mec*, *ccr*, and major differences in junkyard regions. Antimicrob Agents Chemother. 2007; **51**(1): 264-74.
5. Oliveira DC, de Lencastre H. Multiplex PCR strategy for rapid identification of structural types and variants of the *mec* element in methicillin-resistant *Staphylococcus aureus*. Antimicrob Agents Chemother. 2002; **46**(7): 2155-61.
6. McDonald RR, Antonishyn NA, Hansen T, Snook LA, Nagle E, Mulvey MR, et al. Development of a triplex real-time PCR assay for detection of Panton-Valentine leukocidin toxin genes in clinical isolates of methicillin-resistant *Staphylococcus aureus*. Journal of clinical microbiology. 2005; **43**(12): 6147-9.
7. Andrews JM, Testing BWPoS. BSAC standardized disc susceptibility testing method (version 8). J Antimicrob Chemother. 2009; **64**(3): 454-89.
8. Galtier N, Gouy M, Gautier C. SEAVIEW and PHYLO_WIN: two graphic tools for sequence alignment and molecular phylogeny. Comput Appl Biosci. 1996; **12**(6): 543-8.
9. Aanensen DM. Multi Locus Sequence Typing. [Online] [cited 2009 February]; Available from: http://www.mlst.net/
